# Quercetin inhibits LPS-induced macrophage migration by suppressing the iNOS/FAK/paxillin pathway and modulating the cytoskeleton

**DOI:** 10.1080/19336918.2018.1486142

**Published:** 2018-08-01

**Authors:** Shuna Cui, Qingqing Wu, Juan Wang, Min Li, Jing Qian, Shihua Li

**Affiliations:** a Jiangsu Key Laboratory of Integrated Traditional Chinese and Western Medicine for Prevention and Treatment of Senile Diseases, Department of Integrated Chinese and Western Medicine, Medical College of Yangzhou University, Yangzhou, China; b Department of Gynecology and Obstetrics, Affiliated Hospital of Yangzhou University, Yangzhou, China; c Jiangsu Co-Innovation Center for Prevention and Control of Important Animal Infectious Diseases and Zoonoses, Yangzhou, China

**Keywords:** Quercetin, macrophage, migration, iNOS/FAK-paxillin pathway

## Abstract

The natural flavonoid quercetin has antioxidant, anti-inflammatory, and anticancer effects. We investigated the effect of quercetin on lipopolysaccharide (LPS)-induced macrophage migration. Quercetin significantly attenuated LPS-induced inducible nitric oxide synthase (iNOS)-derived nitric oxide (NO) production in RAW264.7 cells without affecting their viability. Additionally, quercetin altered the cell size and induced an elongated morphology and enlarged the vacuoles and concentrated nuclei. Quercetin significantly disrupted the F-actin cytoskeleton structure. Furthermore, quercetin strongly inhibited LPS-induced macrophage adhesion and migration in a dose-dependent manner. Moreover, quercetin inhibited the LPS-induced expression of p-FAK, p-paxillin, FAK, and paxillin as well as the cytoskeletal adapter proteins vinculin and Tensin-2. Therefore, quercetin suppresses LPS-induced migration by inhibiting NO production, disrupting the F-actin cytoskeleton, and suppressing the FAK–paxillin pathway. Quercetin may thus have potential as a therapeutic agent for chronic inflammatory diseases.

## Introduction

Macrophages play an essential role in innate immunity. Macrophages are activated by pathogen-associated molecular patterns (PAMPs) on pathogens or cell debris [1]. Activated macrophages secrete various inflammatory mediators, including nitric oxide (NO) and cytokines, which results in recruitment and activation of immune cells and communication with adaptive immunity by antigen presentation [2]. The recruitment of inflammatory cells to the infection site is vital for host defense. Disruption of this process can result in development of diseases, such as cancer, chronic inflammatory conditions, sepsis, atherosclerosis, and autoimmune disorders [3]. Therefore, regulators of macrophage mobility have therapeutic potential for chronic inflammatory diseases and cancer.

Focal adhesion kinase (FAK) is an intracellular tyrosine kinase with roles in cell motility, cell migration, and cell survival [4]. Indeed, FAK is required for macrophage motility. FAK expression is low in blood monocytes but increases in differentiated macrophages [5,6]. Loss of FAK expression reduces the motility of macrophages [7]. FAK activation is mediated by lipopolysaccharides (LPSs) and dependent on iNOS [3]. The phosphorylation of FAK by the steroid receptor coactivator family of protein tyrosine kinase (Src) at several tyrosine residues, including Tyr 925, affects formation of a complex with paxillin and vinculin and activates the downstream pathways (*e.g*., MAPK cascades) that control cellular morphology and motility [8–12]. Paxillin is a cytoskeletal protein that co-localizes with FAK at focal adhesion contacts [13]. Paxillin mediates cell movement by recruiting cytoskeletal elements and signaling molecules involved in cell attachment, spreading, and migration [14]. The FAK/paxillin interaction is modulated by the cytoskeletal adapter protein vinculin via the extracellular signal-regulated kinase (ERK) pathway [15]. Inhibition of FAK/paxillin in macrophages could prevent the development of inflammatory diseases and cancer [16,17].

Quercetin (Que; 3, 5, 7, 3, 3[3-pentahydroxyflavone]), which is a flavonoid present in fruits and Chinese herbs, has antioxidant [18] and anti-inflammatory effects [19,20]. Studies of the anti-inflammatory activity of quercetin have focused on inhibition of the NF-κB pathway, generation of proinflammatory cytokines, and release of granular components [21,22]. However, the effects of quercetin on LPS-mediated macrophage motility are unclear. Thus, in the current study we investigated the effect of quercetin on NO production and migration by LPS-activated macrophages. The results showed that quercetin inhibited LPS-induced macrophage migration by inhibiting NO production, modulating the F-actin cytoskeleton structure, and inhibiting the FAK–paxillin cascade.

## Results

### Effect of quercetin on macrophage viability

**Figure 1.**
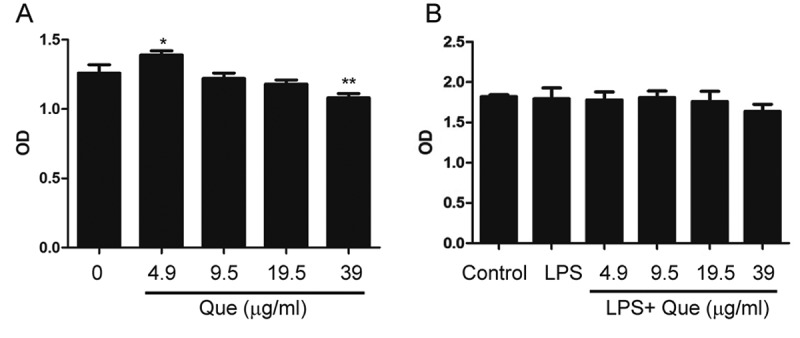
Effects of quercetin on macrophage viability with or without LPS stimulation. (a) Macrophages were incubated with 4.9–39 μg/mL quercetin for 24 h. (b) Macrophages were incubated with 4.9–39 μg/mL quercetin and stimulated with LPS (1 μg/mL) for 24 h. Data are means of three replicates; * p < 0.05, ** p < 0.01 compared with the control.

Only the highest concentration of quercetin alone (39 μg/mL) reduced macrophage viability for 24h treatment; when administered together with LPS (1 μg/mL) for 24h, LPS treatment did not exert a toxic effect on macrophages. Moreover, Quercetin at 4.9–39 μg/mL did not affect the viability of LPS-stimulated macrophages. Therefore, 4.9–39 μg/mL quercetin was used in subsequent experiments (Figure 1).

### Effect of quercetin on NO production

**Figure 2.**
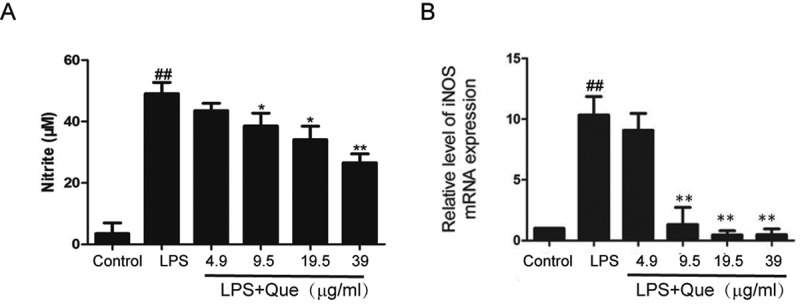
Influence of quercetin on NO production by LPS-stimulated RAW264.7 macrophages. (a) NO production by Griess Reagent; (b) iNOS expression by qPCR. Data are means of three replicates; experiments were performed in triplicate. ## p < 0.01 compared with the control; ** p < 0.01; * p < 0.05 compared with LPS treatment.

NO production was detected by Griess Reagent by measuring nitrite concentration in the culture medium. We observed that LPS (1 μg/mL) strongly stimulates NO production in macrophages after incubation for 24h without affecting the cell viability. Moreover, pretreatment of macrophages with quercetin (9.5–39 µg/mL) for 24h reduced LPS-induced NO release in a concentration-dependent manner (Figure 2(a–b). Cell viability was not affected by quercetin for 24h treatment (Figure 1(b), suggesting that the quercetin-mediated inhibition of LPS-induced NO production was not due to cytotoxicity. Moreover, quercetin at 9.5–39 µg/mL ameliorated the LPS-induced increase in the iNOS mRNA level after 5h treatment.

### Quercetin influences macrophage morphology after LPS stimulation

**Figure 3.**
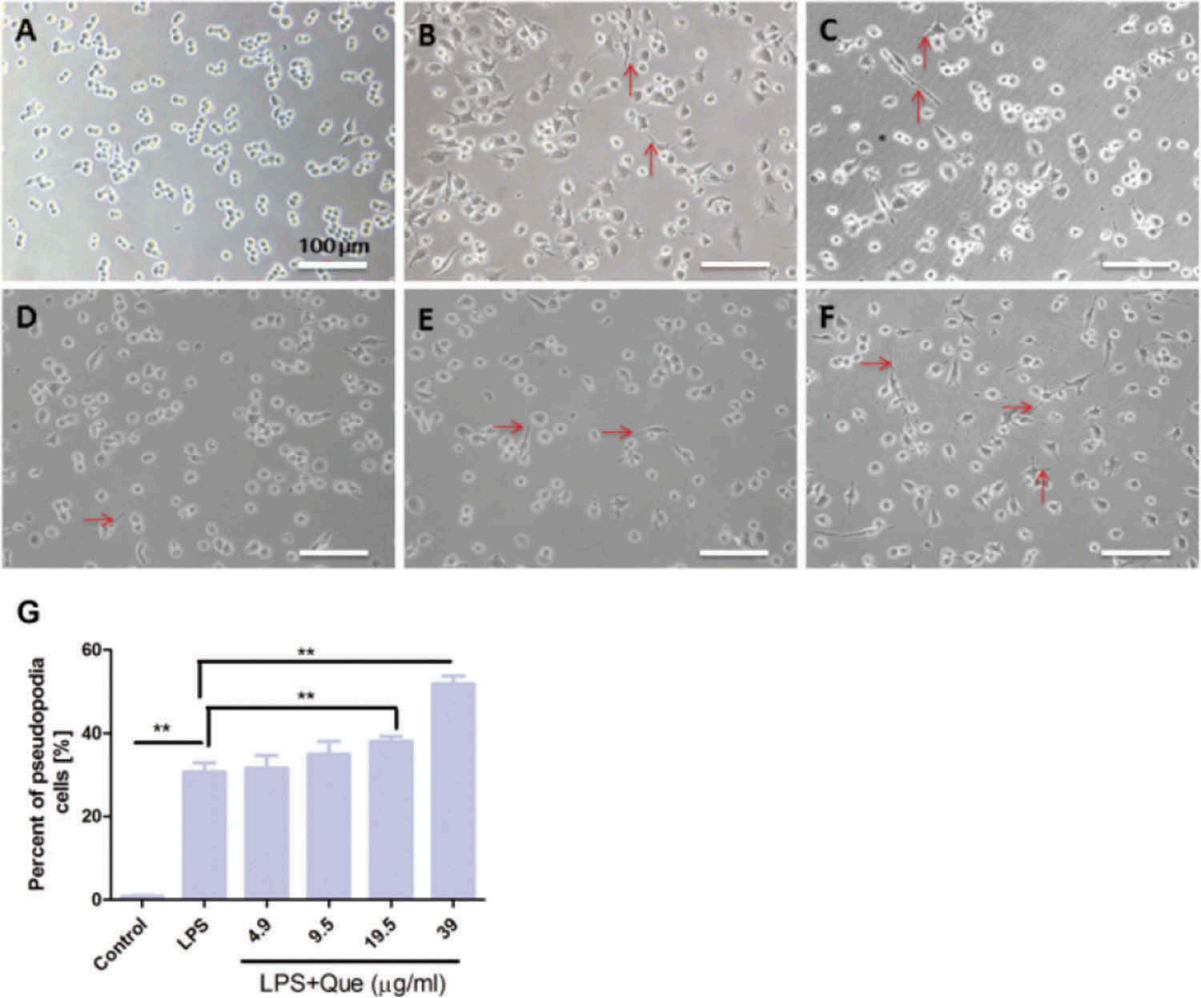
Effect of LPS and quercetin treatment on the morphology of RAW264.7 macrophages. Scale bar, 100 μm. (a) Control. (b) LPS (1 μg/mL); (c) LPS+ Que 4.9 μg/mL; (d) LPS+ Que 9.5 μg/mL; (e) LPS+ Que 19.5 μg/mL; (f) LPS+ Que 39 μg/mL. Magnification, 20×. Red arrow, morphological change. (g). Percentage of cells with pseudopodia in three randomly selected fields. The experiments were performed in triplicate. ** p < 0.01.

Quercetin influenced the morphology of LPS-treated RAW264.7 macrophages. LPS and quercetin treatment caused aberrations in cell morphology in a dose-dependent manner (Figure 3). After 24 h, the control cells were confluent and round, whereas the LPS-treated cells were attached, of increased size, and formed long, slim pseudopodia-like protrusions (Figure 3(b)). Addition of quercetin enhanced the severity of these morphological changes (Figure 3(c–f)). The percentages of pseudopodia cell were significantly increased after LPS treatment, However, quercetin enhanced the percentages of pseudopodia cell in a dose dependent manner compared with LPS treatment (Figure 3(g)).

**Figure 4.**
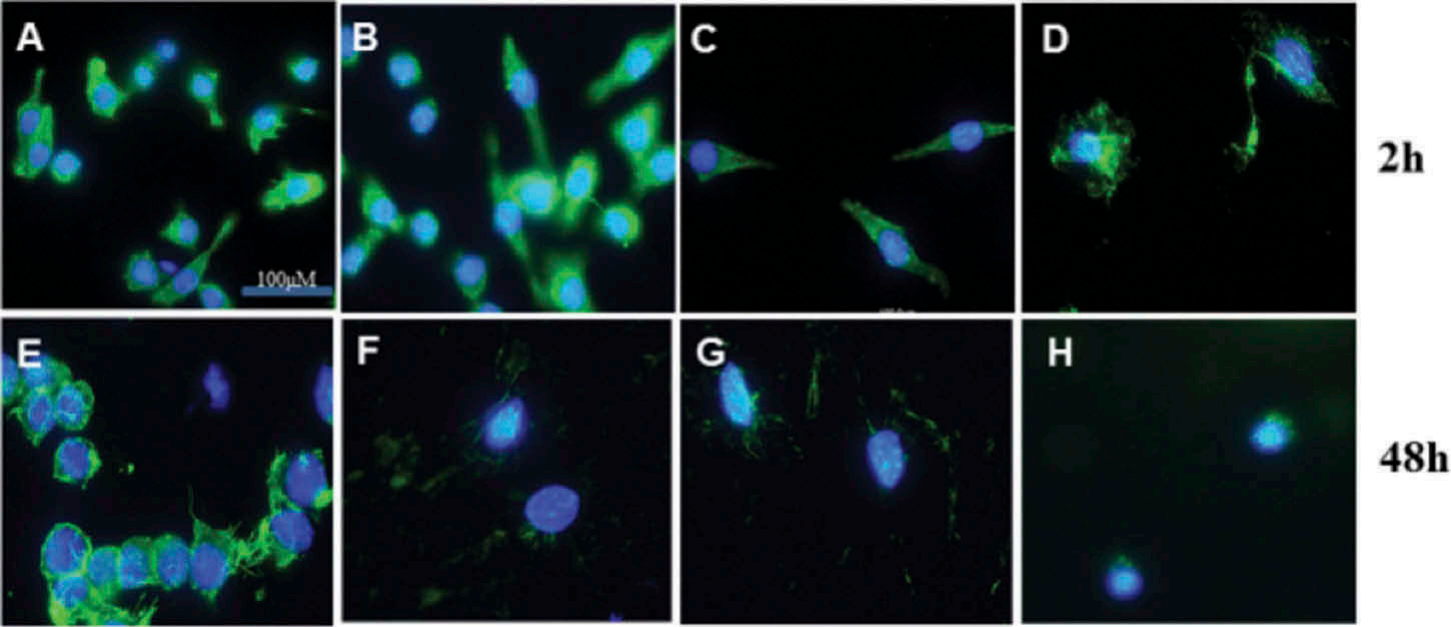
Fluorescence micrographs of the F-actin cytoskeleton of RAW264.7 macrophages after treatment with quercetin for 2 or 48 h. (a) Control; (b) Que 0.4 μg/mL; (c) Que 4 μg/mL; (d) Que 37 μg/mL. Scale bar, 100 μm. Magnification, 100×.

The cytoskeleton plays a vital role in cell movement and morphology. We reported previously that quercetin disrupts F-actin in L929 cells [23]; therefore, we investigated the effect of quercetin on cytoskeleton structure. After incubation for 2 h, quercetin (37 μg/mL) altered the actin cytoskeleton (Figure 4(a–d)). The F-actin structure exhibited marked changes after treatment for 48 h (Figure 4(e–h)); in contrast, actin filaments were evident at the periphery of the control cells.

**Figure 5.**
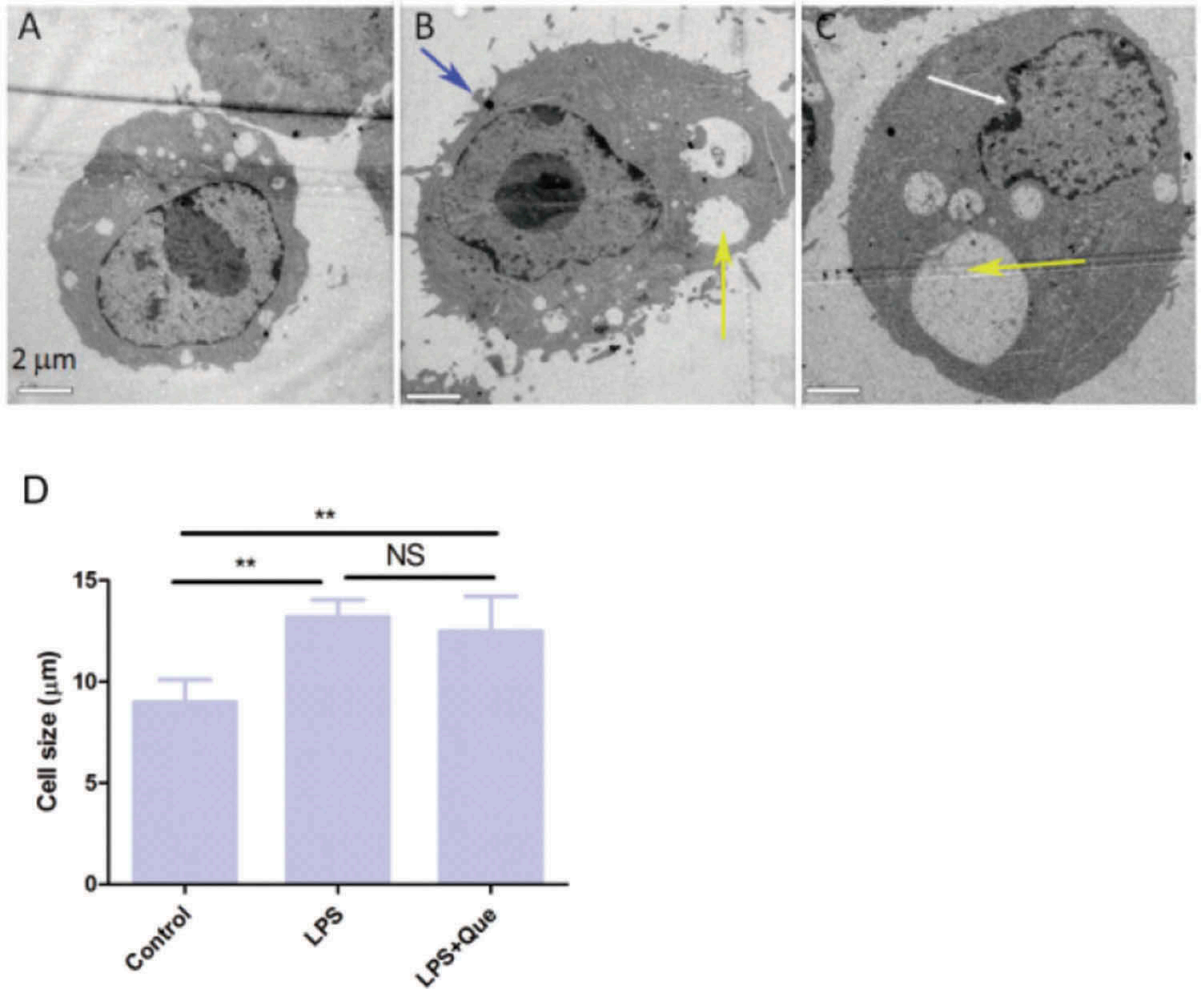
TEM images of macrophages treated with LPS (1 μg/mL) and quercetin (9.5 μg/mL) for 24 h. (a) Control; (b) LPS; (c) LPS+ Que 9.5 μg/mL. Scale bar, 2 μm. Magnification, 1,200×. (d) Cell size was calculated by measuring the diameter of cells in four randomly selected fields.

Regarding ultra-microstructural changes, untreated macrophages were small and round, with a smooth cell membrane and small vacuoles (Figure 5(a)). LPS treatment for 24 h resulted in a significant increase in the size of the macrophages and the formation of cell-surface protrusions (Figure 5(b), blue arrow). Macrophages formed larger phagocytic vacuoles than the untreated macrophages (Figure 5(b), yellow arrow) and had a significantly increased number of Golgi apparati. After quercetin treatment, the cell size and the volume of the phagocytic bulbs increased compared to those of LPS-treated cells (Figure 5(c), yellow arrow), and the nuclear membranes were folded and concentrated with fractured chromosomes (Figure 5(c), white arrow). Therefore, LPS significantly increased the cell size; however, quercetin did not result in a further increase in cell size (Figure 5(d)).

### Quercetin inhibits macrophage adhesion after LPS stimulation

**Figure 6.**
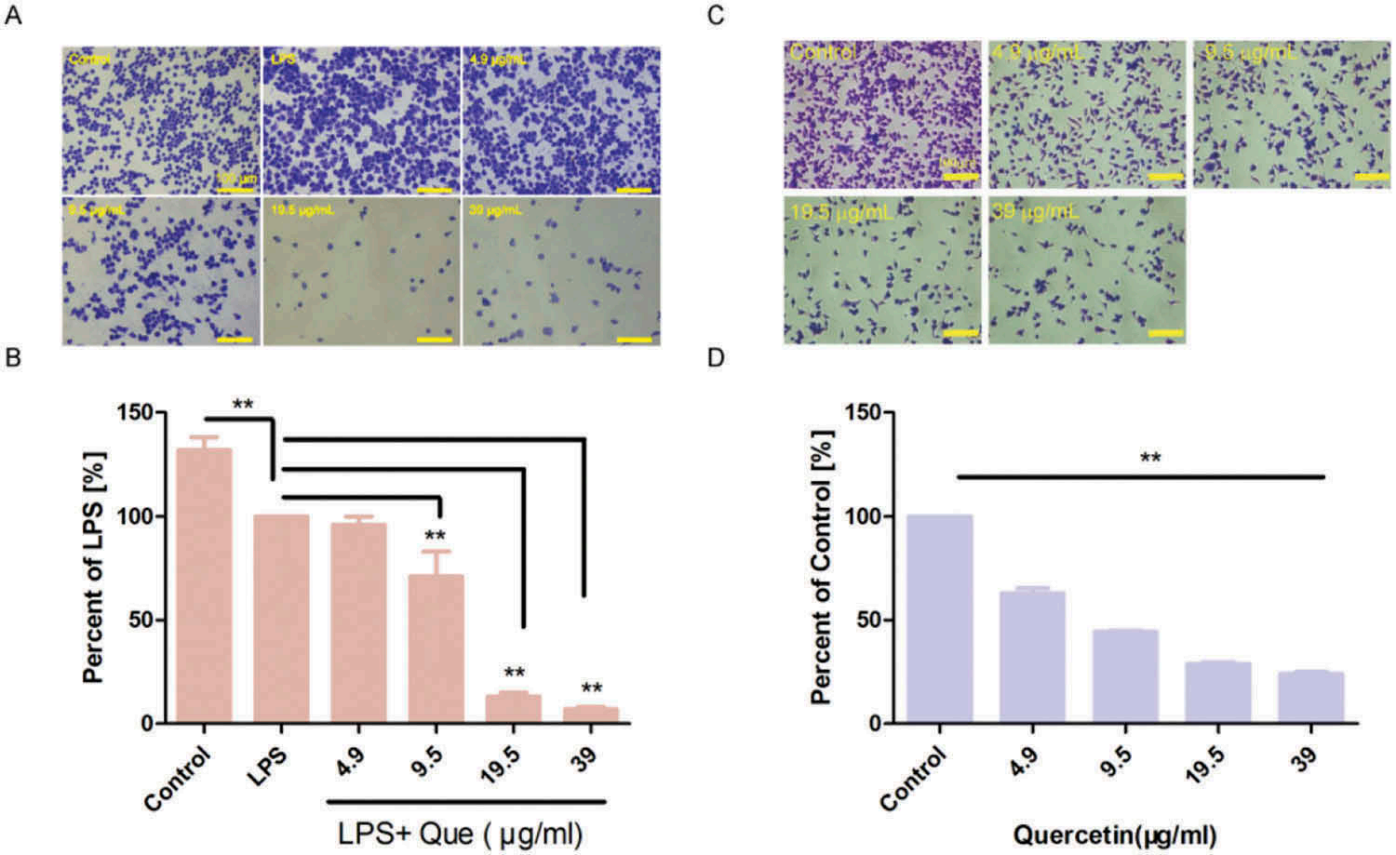
Quercetin inhibits the adhesion of RAW264.7 macrophages. (a) Cells were incubated with or without LPS and quercetin for 24 h; unattached cells were removed; and attached cells were fixed in 4% PFA and stained with crystal violet solution for 10 min at room temperature. (b) Percentage of adherent cells compared to LPS treatment. The experiment was performed in triplicate. ** p < 0.01. (c) Cells were incubated with quercetin for 24 h; unattached cells were removed; and attached cells were fixed in 4% PFA and stained with crystal violet solution for 10 min at room temperature. (d) Percentage of adherent cells compared to the control. The experiment was performed in triplicate. ** p < 0.01 compared with the control.

We investigated the effect of LPS and quercetin on cell adhesion (Figure 6(a–b)). Pretreatment of macrophages with LPS and quercetin for 24 h significantly inhibited their adhesion in a dose-dependent manner (4.9–39 μg/mL), compared with LPS treatment. Moreover, quercetin alone treatment inhibited macrophage adhesion in a dose-dependent manner, compared to the control macrophages (Figure 6(c–d)).

### Quercetin inhibits LPS-induced macrophage migration

**Figure 7.**
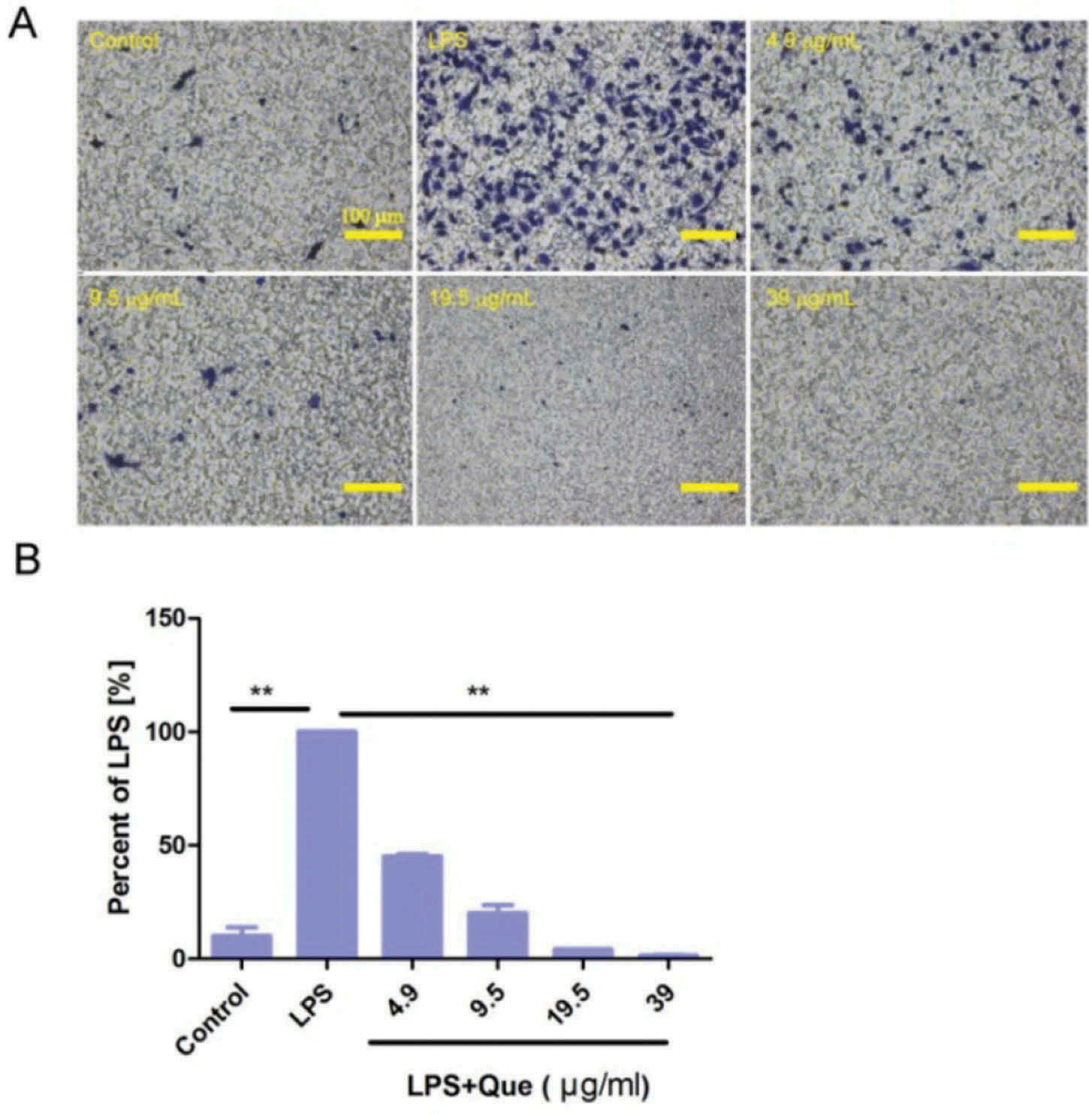
Quercetin suppressed the *in vitro* migration of LPS-stimulated macrophages. (a) Cells were seeded on membranes and incubated with or without LPS and quercetin for 24 h. Cells on the lower surface of the membrane were stained with crystal violet and photographed under a light microscope (magnification, ×100). Results are from three independent experiments. Scale bar, 100 μm. (b) Percentage of migratory cells compared to LPS treatment. The experiment was performed in triplicate. ** p < 0.01 compared with LPS as a control.

In Transwell assays, untreated macrophages showed little migration. However, LPS-treated macrophages showed significantly increased migration. Quercetin (4.9–39 μg/mL) treatment significantly inhibited macrophage migration in a dose-dependent manner, respectively, compared with LPS-treated cells (Figure 7(c–f)).

### Quercetin modulates inos and fak-paxillin protein levels

**Figure 8.**
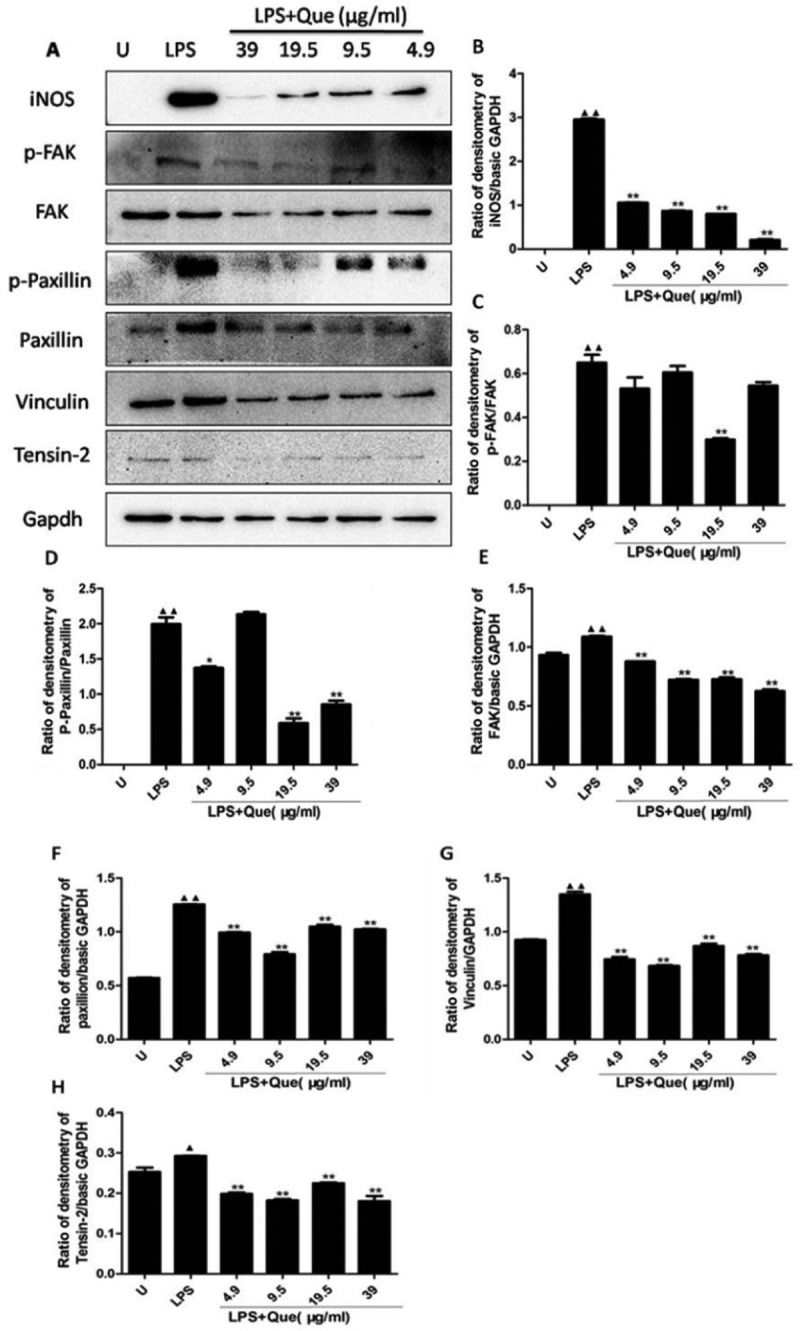
Effect of quercetin on iNOS and FAK-paxillin protein levels in macrophages. Total cell lysates were prepared after treatment with quercetin or LPS for 24 h. (a) Western blotting for iNOS and FAK-paxillin proteins. Results are representative of three independent experiments. (b–h) Band intensity determined using Image J software. * p < 0.05, ** p < 0.01 compared with LPS treatment. ^▲^ *p *< 0.05, ^▲▲^ *p *< 0.01, compared with no treatment.

Because quercetin significantly inhibited NO production and *iNOS* expression in LPS-treated macrophages (Figure 2(a–b)), we determined the iNOS protein level in LPS-treated macrophages. Quercetin at 4.9–39 μg/mL decreased the iNOS protein level in a dose-dependent manner (Figure 8(b)).

Quercetin inhibited the migration of macrophages, and the FAK-paxillin pathway is associated with cell motility [24–26]. Thus, we investigated whether quercetin inhibited cell migration by suppressing the production of proteins involved in the FAK-paxillin pathway (Figure 8(a,c–f)). Untreated macrophages express FAK and low level of paxillin protein. LPS treatment increased the p-FAK^Tyr925^, FAK, p-paxillin^Tyr118^, and paxillin protein levels compared with the untreated control macrophages. Addition of quercetin significantly reduced the magnitude of the LPS-induced increase in the p-FAK^Tyr25^, FAK, p-paxillin^Tyr18^, and paxillin protein levels. Moreover, LPS increased the vinculin and tesin-2 protein levels, which were regulated by quercetin (Figure 8(g–h)). Therefore, the effect of quercetin on the migration of LPS-induced macrophages is mediated by its modulation of the FAK-paxillin pathway.

### Quercetin regulates the FAK and paxillin protein levels

**Figure 9.**
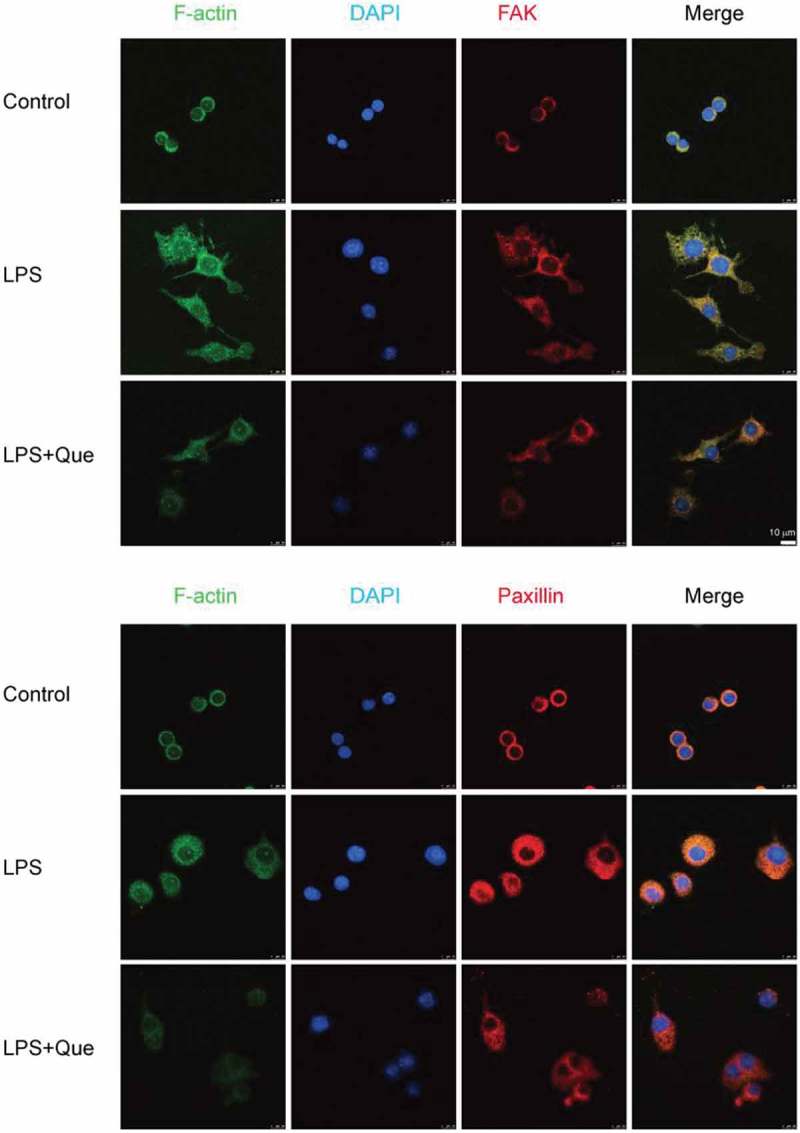
Modulation of FAK and paxillin expression in LPS-induced macrophage by quercetin. RAW264.7 macrophages were treated with or without LPS and quercetin (9.5 μg/mL) for 24 h. The cells were fixed and subjected to immunofluorescence for FAK and paxillin (red), F-actin (green), and nuclei (DAPI, blue) staining. Scale bar, 10 μm.

Immunofluorescence staining showed that LPS increased FAK and paxillin expression, whereas quercetin treatment decreased their expression at the cell periphery, Moreover, LPS increased the expression of F-actin structure in macrophages cells, whereas, quercetin strongly decreased the F-actin intensity compared with LPS treatment (Figure 9).

## Discussion

Quercetin suppresses the LPS-induced inflammatory response via various mechanisms [18,22,27,28]; *e.g*., by inhibiting the NF-κB, STAT-1, and Syk/Src/IRAK-1 pathways. However, little is known about the effect of quercetin on cell motility. Macrophages play an important role in innate immunity, and their recruitment to sites of infection or injury is important for controlling infection and tissue repair. However, uncontrolled or abnormal accumulation of monocytes can lead to development of cancer or chronic inflammation. Therefore, we assessed the effect of quercetin on LPS-induced macrophages, focusing on their motility.

In accordance with our previous report [23], quercetin alone at 39 μg/mL reduced the viability of macrophages for 24h treatment by WST-8 assay. However, quercetin inhibited LPS-induced NO release by macrophages without affecting their viability. Moreover, quercetin reduced the LPS-induced iNOS production at the protein and mRNA levels. Quercetin exerts strong effects on LPS induced iNOS mRNA levels compared with NO production in the culture medium. This may due to the sensitivity of detection methods. Griess reagent detected the Nitrite level in the culture medium, which is one of two primary stable and nonvolatile breakdown products of nitric oxide. However, RT-PCR detected the iNOS mRNA expression in whole cells. This may lead to different pattern of quercetin effect. Sustained production of NO can lead to inflammation and tumorigenesis. NO release stimulates migration of several types of cells, such as primary aortic smooth muscle cells [29] and epithelial cells [30]. Moreover, LPS induces macrophage migration through the Src-FAK cascade, which is highly dependent on iNOS [3,12]. Therefore, inhibition of NO release could be a mechanism by which quercetin inhibits the migration of LPS-stimulated macrophages.

We next investigated whether the inhibitory effects of quercetin on LPS-induced expression of iNOS is associated with the decreased cell migration and FAK-paxillin expression. Cell migration is essential for angiogenesis and wound healing. It involves formation from the plasma membrane of filopodia or lamellipodia, which are supported by actin filaments and associated proteins [14]. Quercetin altered macrophage morphology by inducing some cells to form irregular shapes (*e.g*., diamond) or produce slim pseudopodia-like protrusions. The morphology of intracellular structures, such as vacuoles and nuclei, was also influenced by quercetin. Moreover, quercetin disrupted the structure of F-actin and reduced FAK and paxillin protein levels at focal adhesion contacts at the leading edges of macrophages. Therefore, quercetin impairs the formation of focal adhesions and actin bundles by suppressing the production of FAK and paxillin. This is consistent with a previous report that blocking the expression or function of FAK and paxillin reduced cell mobility [31]. Moreover, we previously reported that quercetin attenuates the phagocytosis of *Candida albicans* by macrophages by disrupting the F-actin cytoskeleton structure [23]. Moreover, quercetin can directly bind to actin [32,33]. Therefore, quercetin likely inhibits cell migration by altering the cytoskeleton.

We next evaluated the effect of LPS and quercetin on adhesion of macrophages. Quercetin inhibited macrophage adhesion in a dose-dependent manner, whereas LPS had no effect. This may be because the larger size of the LPS-treated cells resulted in fewer cells being able to attach to the same surface area compared to untreated cells. Moreover, LPS treatment increased cell motility; moving cells are less tightly attached to the substrate. These results confirm that quercetin disrupted the cytoskeleton and decreased cell motility.

The FAK pathway is reportedly important in LPS-activated macrophages [11]. We observed that untreated macrophages express FAK, the results of this study were consistent with the other study that expression of FAK is increased in differentiated macrophages [6]. Moreover, quercetin inhibited the LPS-induced expression of p-FAK, FAK, p-paxillin, and paxillin, as well as the vinculin and tensin-2 protein levels, in a concentration-independent manner. Therefore, quercetin influences LPS-induced cell migration by modulating the FAK/paxillin pathway. These results are in agreement with our *in vitro* finding that, in the presence of quercetin, LPS induces macrophage migration.

In conclusion, quercetin inhibits LPS-induced NO production, disrupts the F-actin cytoskeleton, and inhibits macrophage migration. Because abnormal accumulation of macrophages can contribute to disease progression (*e.g*., cancer and chronic inflammatory diseases), our data suggest that iNOS/FAK are targets of quercetin in the presence of LPS-induced inflammation. Furthermore, quercetin could be used to control infection and chronic inflammation, as well as to maintain tissue homeostasis.

## Materials and methods

### Materials

Dulbecco’s modified Eagle’s Medium (DMEM), 100× penicillin and streptomycin, and fetal bovine serum (FBS) were from Lonza (Verviers, Belgium). Dimethyl sulfoxide (DMSO), lipopolysaccharide (LPS), quercetin, NaNO_2,_ sulfanilamide, naphthylethylene diamine dihydrochloride, and phosphoric acid were purchased from Sigma-Aldrich (St. Louis, MO, USA). The WST-8 assay kit and phosphatase and protease inhibitor cocktail set I were from Biotool (Shanghai, China). Primary and secondary antibodies (Table 1) for Western blotting and immunofluorescence were purchased from Cell Signaling Technology (Danvers, MA,USA) and Abcam (Shanghai, China).

**Table 1.** Antibodies used in this study.

	Name	Source	ID	Usage
1	Paxillin	Rabbit, pAb	#2542 cell signaling	WB
2	Anti-rabbit IgG, HRP-linked antibody	Goat	#7074 cell signaling	WB
3	Phospho–Paxillin (Tyr118)	Rabbit, pAb	#2541 cell signaling	WB
4	FAK	Rabbit, pAb	#13430 cell signaling	WB
5	Tensin 2	Rabbit, pAb	#13430 cell signaling	WB
6	Vinculin	Rabbit, pAb	#13430 cell signaling	WB
7	Phospho-FAK(Tyr925)	Rabbit, pAb	#9330 cell signaling	WB
8	GAPDH-HRP	Mouse, mAb	#ab011 Multi Science	WB
9	Anti-FAK antibody [EP695Y]	Rabbit, mAb	#ab40794abcam	IF
10	Anti-paxillin antibody [Y113]	Rabbit, mAb	#ab32084abcam	IF
11	Anti-rabbit IgG (H + L), F(ab’)2 Fragment (Alexa Fluor® 555 Conjugate)	Goat	#4413cell signaling	IF
12	CytoPainter Phalloidin-iFluor 488 Reagent		#ab176753abcam	IF
13	DAPI staining buffer		#C1005Beyotime	IF

### Cell culture

RAW 264.7 murine macrophages were purchased from the Type Culture Collection of the Chinese Academy of Sciences, Shanghai, China. The cells were cultured in DMEM supplemented with 10% FBS at 37°C in a 5% CO_2_ atmosphere.

### WST-8 assay

WST-8 assays were performed as reported previously [34]. RAW267.4 macrophages were seeded into 96-well cell culture plates at 5 × 10^5^ cells/mL in DMEM containing the indicated concentrations of quercetin with or without LPS (1 μg/mL) for 24 h. Next, 10 μL WST-8 per well were added, and the plate was placed on a plate shaker for 1 min to ensure optimal mixing. After incubation for 30 min, the optical density (OD) at 450 nm was determined using a microtiter plate reader (Bio-Rad, Hercules, CA). The mean and standard deviation of three replicates were determined.

### Cell morphology

RAW267.4 macrophages were seeded into 96-well cell culture plates at 5 × 10^5^ cells/mL in DMEM containing the indicated concentrations of quercetin with or without LPS (1 μg/mL) for 24 h. Images of three randomly selected fields were obtained using an inverted microscope. The percentage of cells with pseudopodia was calculated using Image J software as the number of cells with pseudopodia ÷ the total number of cells ×100%.

### Nitric oxide assay

The NO concentrations in cell culture supernatants were determined by measuring the accumulation of nitrite (NO_2_^−^) [29]. Briefly, 100 μL of Griess reagent (1% sulfanilamide, 0.1% naphthylethylene diamine dihydrochloride, and 2.5% phosphoric acid) were mixed with an equal volume of culture supernatant in 96-well flat-bottomed microplates and incubated at room temperature for 10 min. The OD at 540 nm was read using a microtiter plate reader. Nitrite concentrations were determined from a standard curve established using serial dilutions of NaNO_2_.

### Adhesion assay

Adhesion assays were performed as reported previously [35]. Cells were plated in 12-well plates at 5 × 10^5^ cells/mL for 12 h and subsequently incubated with quercetin or LPS (1 μg/mL) for 24 h. Cells were harvested and reseeded in 24-well plates for 3 h. Cells were washed and fixed with 4% PFA at room temperature for 15 min. Cristal violet staining solution (Beyotime, Beijing, China) was next added, and the plates were incubated for 10 min at room temperature. Images of five randomly selected fields were obtained using an inverted microscope. Cells were enumerated using Image J software. The percentage of adhesion compared to LPS treatment or control group was calculated.

### Transwell assay

Transwell assays were performed in triplicate as reported previously [35]. Cells (4 × 10^4^/well) were placed in Transwell^R^ cell culture chambers (8 mm pore size; Corning, Lowell, MA, USA). Cell suspension was placed in the upper chamber of a Transwell^R^ insert and incubated with the indicated concentrations of quercetin and DMSO in serum-free DMEM. The lower chamber was filled with DMEM containing 20% FBS as a chemoattractant, and the system was incubated under the normal culture condition for 24 h. The lower surface of the membrane was treated with 4% PFA and stained with crystal violet. Cells in five random fields per chamber were counted using an inverted microscope and Image J software. The percentage of migration was calculated and compared to that under LPS treatment.

### Transmission electron microscopy

Transmission electron microscopy (TEM) was performed as described previously [36]. Briefly, macrophages (1 × 10^6^ cells/mL) were incubated for 24 h with or without LPS and quercetin (9.5 μg/mL) in six-well plates. The cells were next harvested and fixed in 2.5% glutaraldehyde for 2 h at 4°C. Samples were subsequently rinsed with 0.1 M PBS, fixed in 1% osmium tetroxide for 1–2 h, and dehydrated sequentially in 50, 70, 80, 90, and 100% ethanol for 15 min. The specimens were embedded in Epon™ and observed by TEM (CM100, Philips, The Netherlands). Cell size was calculated using Image J software by measuring cell diameters in four randomly selected fields.

### F-actin cytoskeleton staining

Immunofluorescence staining for F-actin was performed as reported previously [23]. Macrophages (1 × 10^5^/mL) were seeded in a four-well plates with/without quercetin (0.4, 4, and 37 μg/mL) and incubated for 2 and 48 h under standard conditions. The cells were washed and fixed with 3.7% PFA for 15 min at room temperature and then washed and permeabilized with 0.1% Triton X in PBS for 5 min. The cells were washed and incubated with Alexa Fluor® 488-phalloidin for 1 h, subsequently stained with DAPI (1 μg/mL), and visualized using a BZ-8000 digital fluorescence microscope equipped with BZ-analysis software (Keyence GmbH, Neu-Isenburg, Germany).

### Immunofluorescence staining for FAK and paxillin

Immunofluorescence assays were performed as reported previously [23,35]. Briefly, cells (3 × 10^5^/mL) were incubated for 24 h with or without LPS (1 μg/mL) and 19.5 μg/mL quercetin and then fixed in 4% paraformaldehyde (PFA) for 20 min at room temperature. The cells were permeabilized by incubating with 0.1% Triton X-100 in PBS for 15 min. Subsequently, the cells were blocked by incubating with 2% BSA for 1 h and incubated with primary antibodies against FAK (1:500) and paxillin (1:500) overnight at 4°C; this was followed by incubation with anti-rabbit IgG secondary antibodies (1:500) and iFluor 488-conjugated phalloidin (1:1000) (Abcam) for 1 h at room temperature; 100 μL of DAPI solution (Beyotime, Beijing, China) was added to stain nuclei. Samples were examined using a TCS SP8 STED ultra-high-resolution laser confocal microscope (Leica, Japan).

### Western blotting

Cells were pretreated with or without LPS (1 μg/mL), DMSO (control), or quercetin (4.9–39 μg/mL) for 24 h. Cells were washed with ice-cold PBS, scraped and stored at −20°C. Cell lysates were prepared in ice-cold lysis buffer and clarified by centrifugation. Protein concentrations were quantified by BCA assay. Proteins (20 μg) were resolved by 10% sodium dodecyl sulfate-polyacrylamide gel electrophoresis (SDS-PAGE) and transferred onto polyvinylidene difluoride (PVDF) membranes (Bio-Rad) by electroblotting at 100 V for 90 min. Membranes were probed with the appropriate primary antibodies (1:1,000 dilution) followed by an HRP-conjugated secondary antibody (1:3,000 dilution); bands were detected by enhanced chemiluminescence.

### Quantitative PCR

Quantitative PCR (qPCR) was performed as reported previously [35]. Briefly, cells were pretreated with LPS (1 μg/mL) or DMSO (control) and quercetin (4.9–39 μg/mL) for 5 h, and total RNA was extracted using the RNeasy Plus Mini Kit (Qiagen, Valencia, CA). cDNA was prepared using a reverse transcription reagent kit (Selleck Company, Shanghai, China) according to the manufacturer’s instructions. Target genes were amplified using the Light Cycler® 96 Real-Time PCR System (Roche, Indianapolis, IN) with the following specific primers: iNOS, 5ʹ-CCTTGTTCAGCTACGCCTTC-3ʹ (forward) and 5ʹ-CTGAGGGCTCTGTTGAGGTC-3ʹ (reverse); GAPDH, 5ʹ-GCACCGTCAAGGCTGAGAAC-3ʹ(forward) and 5ʹ-TGGTGAAGACGCCAGTGGA-3ʹ(reverse). GAPDH was utilized for normalization. Each sample was amplified in triplicate, and expression levels were normalized using the 2^−ΔΔCt^ method.

### Statistical analysis

Data are presented as means ± SD of at least three experiments. A value of p < 0.05 by Student’s *t*-test was taken to indicate statistical significance.

## References

[1] Abbas AK, Janeway CA Jr. Immunology: improving on nature in the twenty-first century. Cell. 2000;100:129–138. 10647937 10.1016/s0092-8674(00)81689-x

[2] Bock FJ, Chang P. Macrophage activation: on par with LPS. Chem Biol. 2015;22:432–433. 25910238 10.1016/j.chembiol.2015.04.006

[3] Maa MC, Chang MY, Chen YJ, et al Requirement of inducible nitric-oxide synthase in lipopolysaccharide-mediated Src induction and macrophage migration. J Biol Chem. 2008;283:31408–31416. 18786925 10.1074/jbc.M801158200

[4] Maa MC, Chang MY, Hsieh MY, et al Butyrate reduced lipopolysaccharide-mediated macrophage migration by suppression of Src enhancement and focal adhesion kinase activity. J Nutr Biochem. 2010;21:1186–1192. 20149623 10.1016/j.jnutbio.2009.10.004

[5] Lin TH, Yurochko A, Kornberg L, et al The role of protein tyrosine phosphorylation in integrin-mediated gene induction in monocytes. J Cell Biol. 1994;126:1585–1593. 8089188 10.1083/jcb.126.6.1585 PMC2290955

[6] Heuslein JL, Murrell KP, Leiphart RJ, et al Vascular growth responses to chronic arterial occlusion are unaffected by myeloid specific focal adhesion kinase (FAK) deletion. Sci Rep. 2016;6:27029. 27244251 10.1038/srep27029 PMC4886679

[7] Owen KA, Pixley FJ, Thomas KS, et al Regulation of lamellipodial persistence, adhesion turnover, and motility in macrophages by focal adhesion kinase. J Cell Biol. 2007;179:1275–1287. 18070912 10.1083/jcb.200708093 PMC2140030

[8] Zhao X, Guan JL Focal adhesion kinase and its signaling pathways in cell migration and angiogenesis. Adv Drug Deliv Rev. 2011;63:610–615. 21118706 10.1016/j.addr.2010.11.001 PMC3132829

[9] Sulzmaier FJ, Jean C, Schlaepfer DD FAK in cancer: mechanistic findings and clinical applications. Nat Rev Cancer. 2014;14:598–610. 25098269 10.1038/nrc3792 PMC4365862

[10] Kim SH, Turnbull J, Guimond S Extracellular matrix and cell signalling: the dynamic cooperation of integrin, proteoglycan and growth factor receptor. J Endocrinol. 2011;209:139–151. 21307119 10.1530/JOE-10-0377

[11] Digiacomo G, Tusa I, Bacci M, et al Fibronectin induces macrophage migration through a SFK-FAK/CSF-1R pathway. Cell Adh Migr. 2017;11:327–337. 27588738 10.1080/19336918.2016.1221566 PMC5569968

[12] Maa MC, Chang MY, Li J, et al The iNOS/Src/FAK axis is critical in Toll-like receptor-mediated cell motility in macrophages. Biochim Biophys Acta. 2011;1813:136–147. 20849883 10.1016/j.bbamcr.2010.09.004

[13] Webb DJ, Donais K, Whitmore LA, et al FAK-Src signalling through paxillin, ERK and MLCK regulates adhesion disassembly. Nat Cell Biol. 2004;6:154–161. 14743221 10.1038/ncb1094

[14] Lopez-Colome AM, Lee-Rivera I, Benavides-Hidalgo R, et al Paxillin: a crossroad in pathological cell migration. J Hematol Oncol. 2017;10:50. 28214467 10.1186/s13045-017-0418-y PMC5316197

[15] Subauste MC, Pertz O, Adamson ED, et al Vinculin modulation of paxillin-FAK interactions regulates ERK to control survival and motility. J Cell Biol. 2004;165:371–381. 15138291 10.1083/jcb.200308011 PMC2172187

[16] Jiamvoraphong N, Jantaratnotai N, Sanvarinda P, et al Concurrent suppression of NF-kappaB, p38 MAPK and reactive oxygen species formation underlies the effect of a novel compound isolated from Curcuma comosa Roxb. LPS-activated Microglia J Pharm Pharmacol. 2017;69:917–924. 28382728 10.1111/jphp.12723

[17] Park SY, Jin ML, Kang NJ, et al Anti-inflammatory effects of novel polygonum multiflorum compound via inhibiting NF-kappaB/MAPK and upregulating the Nrf2 pathways in LPS-stimulated microglia. Neurosci Lett. 2017;651:43–51. 28458018 10.1016/j.neulet.2017.04.057

[18] Li C, Zhang WJ, Frei B Quercetin inhibits LPS-induced adhesion molecule expression and oxidant production in human aortic endothelial cells by p38-mediated Nrf2 activation and antioxidant enzyme induction. Redox Biol. 2016;9:104–113. 27454768 10.1016/j.redox.2016.06.006 PMC4961307

[19] Guo C, Hou GQ, Li XD, et al Quercetin triggers apoptosis of lipopolysaccharide (LPS)-induced osteoclasts and inhibits bone resorption in RAW264.7 cells. Cell Physiol Biochem. 2012;30:123–136. 22759961 10.1159/000339052

[20] Cho YH, Kim NH, Khan I, et al Anti-inflammatory potential of quercetin-3-O-beta-D-(“2”-galloyl)-glucopyranoside and quercetin isolated from diospyros kaki calyx via suppression of MAP signaling molecules in LPS-induced RAW 264.7 macrophages. J Food Sci. 2016;81:C2447–C56. 27648736 10.1111/1750-3841.13497

[21] Peng Z, Gong X, Yang Y, et al Hepatoprotective effect of quercetin against LPS/d-GalN induced acute liver injury in mice by inhibiting the IKK/NF-kappaB and MAPK signal pathways. Int Immunopharmacol. 2017;52:281–289. 28963941 10.1016/j.intimp.2017.09.022

[22] Zhang M, Lin JM, Li XS, et al Quercetin ameliorates LPS-induced inflammation in human peripheral blood mononuclear cells by inhibition of the TLR2-NF-kappaB pathway. Genet Mol Res GMR. 2016;15. 10.4238/gmr.15028297 27421015

[23] Cui S, Qian J, Bo P Inhibitive effect on phagocytosis of Candida albicans induced by pretreatment with quercetin via actin cytoskeleton interference. Jl Traditional Chin Med. 2013;33:804–809. 10.1016/s0254-6272(14)60016-9 24660615

[24] Grimaldi AM, Simeone E, Festino L, et al Novel mechanisms and therapeutic approaches in melanoma: targeting the MAPK pathway. Discov Med. 2015;19:455–461. 26175403

[25] Carlino MS, Long GV, Kefford RF, et al Targeting oncogenic BRAF and aberrant MAPK activation in the treatment of cutaneous melanoma. Crit Rev Oncol Hematol. 2015;96:385–398. 26358420 10.1016/j.critrevonc.2015.08.021

[26] Deramaudt TB, Dujardin D, Noulet F, et al Altering FAK-paxillin interactions reduces adhesion, migration and invasion processes. PLoS One. 2014;9:e92059. 24642576 10.1371/journal.pone.0092059 PMC3958421

[27] Park JY, Lim MS, Kim SI, et al Quercetin-3-O-beta-D-glucuronide suppresses lipopolysaccharide-induced JNK and ERK phosphorylation in LPS-challenged RAW264.7 cells. Biomol Ther (Seoul). 2016;24:610–615. 27257013 10.4062/biomolther.2016.026 PMC5098540

[28] Yang WS, Jeong D, Yi YS, et al Myrsine seguinii ethanolic extract and its active component quercetin inhibit macrophage activation and peritonitis induced by LPS by targeting to Syk/Src/IRAK-1. J Ethnopharmacol. 2014;151:1165–1174. 24378351 10.1016/j.jep.2013.12.033

[29] Brown C, Pan X, Hassid A Nitric oxide and C-type atrial natriuretic peptide stimulate primary aortic smooth muscle cell migration via a cGMP-dependent mechanism: relationship to microfilament dissociation and altered cell morphology. Circ Res. 1999;84:655–667. 10189353 10.1161/01.res.84.6.655

[30] Noiri E, Peresleni T, Srivastava N, et al Nitric oxide is necessary for a switch from stationary to locomoting phenotype in epithelial cells. Am J Physiol. 1996;270:C794–802. 8638659 10.1152/ajpcell.1996.270.3.C794

[31] Panetti TS Tyrosine phosphorylation of paxillin, FAK, and p130CAS: effects on cell spreading and migration. Front Biosci. 2002;7:d143–50. 11779709 10.2741/A771

[32] Bohl M, Czupalla C, Tokalov SV, et al Identification of actin as quercetin-binding protein: an approach to identify target molecules for specific ligands. Anal Biochem. 2005;346:295–299. 16213457 10.1016/j.ab.2005.08.037

[33] Bohl M, Tietze S, Sokoll A, et al Flavonoids affect actin functions in cytoplasm and nucleus. Biophys J. 2007;93:2767–2780. 17573428 10.1529/biophysj.107.107813 PMC1989700

[34] Cui S, Wienhoefer N, Bilitewski U Genistein induces morphology change and G2/M cell cycle arrest by inducing p38 MAPK activation in macrophages. Int Immunopharmacol. 2014;18:142–150. 24290959 10.1016/j.intimp.2013.11.016

[35] Cui S, Wang J, Wu Q, et al Genistein inhibits the growth and regulates the migration and invasion abilities of melanoma cells via the FAK/paxillin and MAPK pathways. Oncotarget. 2017;8:21674–21691. 28423510 10.18632/oncotarget.15535 PMC5400615

[36] Wang H, Tao L, Ni T, et al Anticancer efficacy of the ethyl acetate extract from the traditional Chinese medicine herb Celastrus orbiculatus against human gastric cancer. J Ethnopharmacol. 2017;205:147–157. 28476678 10.1016/j.jep.2017.04.030

